# Association of Repeated Antibiotic Exposure Up to Age 4 Years With Body Mass at Age 4.5 Years

**DOI:** 10.1001/jamanetworkopen.2019.17577

**Published:** 2020-01-22

**Authors:** Carol Chelimo, Carlos A. Camargo, Susan M. B. Morton, Cameron C. Grant

**Affiliations:** 1 Department of Paediatrics: Child & Youth Health, University of Auckland, Auckland, New Zealand; 2 Centre for Longitudinal Research–He Ara Ki Mua, University of Auckland, Auckland, New Zealand; 3 Department of Emergency Medicine, Massachusetts General Hospital, Harvard Medical School, Boston; 4 General Paediatrics, Starship Children’s Hospital, Auckland, New Zealand

## Abstract

**Importance:**

Antibiotic exposures in early life may affect weight by altering gut microbiota, potentially increasing the likelihood of childhood obesity.

**Objective:**

To examine whether repeated antibiotic exposure by age 48 months is associated with higher body mass index (BMI) at age 54 months.

**Design, Setting, and Participants:**

This research was undertaken within a prospective cohort study in New Zealand (Growing Up in New Zealand) that recruited 6853 children antenatally during 2009 to 2010. At the 54-month follow-up, 5734 of 6156 children (93%) had their weight and height measured. Community pharmacy antibiotic dispensing data were obtained from the New Zealand Pharmaceutical Collection database for children whose parents consented to external data linkage. The analytic sample comprised singletons with 54-month weight and height measurements, community antibiotic dispensing and birth weight data, gestational age greater than 27 weeks, and no congenital anomalies. Data analysis took place from 2017 to 2018.

**Exposures:**

Antibiotic exposure (yes or no), the number of dispensings, age at first exposure, and timing (age) of exposures between birth and age 48 months.

**Main Outcomes and Measures:**

World Health Organization BMI-for-age *z* scores; and International Obesity Task Force overweight and obesity cutoff points that pass through adult BMI values of 25 and 30.

**Results:**

Of the 5128 singletons (2622 [51%] male; mean [SD] birth weight of 3527 [542] g), 4886 (95%) had an antibiotic prescription by age 48 months and 437 (9%) had obesity at age 54 months. Adjusted mean (SE) BMI-for-age *z* scores increased significantly with the number of antibiotic dispensings for 4 to 6, 7 to 9, and more than 9 dispensings (unexposed, 0.87 [0.09]; 1-3 exposures, 0.92 [0.06] [*P* = .57]; 4-6 exposures, 1.06 [0.06] [*P* = .02]; 7-9 exposures, 1.06 [0.06] [*P* = .02]; >9 exposures, 1.08 [0.05] [*P* = .01]). Additionally, receiving more than 9 dispensings was associated with greater likelihood of obesity compared with no exposure (adjusted odds ratio, 2.41; 95% CI, 1.07-5.41). Children whose exposure began in the first year of life had a higher adjusted mean (SD) BMI-for-age *z* score than those not exposed (1.06 [0.05] vs 0.89 [0.09]; *P* = .03), whereas those whose exposure commenced after the first year of life did not (1.02 [0.06] vs 0.89 [0.09]; *P* = .10).

**Conclusions and Relevance:**

In this study, repeated antibiotic exposure in early childhood was associated with higher mean BMI-for-age *z* score and an increased likelihood of obesity. Future research could examine whether interventions such as antibiotic stewardship programs, which are designed to reduce overprescribing of antibiotics, also reduce early childhood obesity.

## Key Points

**Question:**

Is repeated antibiotic exposure by age 48 months associated with higher body mass index (BMI) and the likelihood of overweight or obesity at age 54 months?

**Findings:**

In this cohort study that included 5128 children, those repeatedly exposed to antibiotics had significantly higher BMI than those not exposed. As the number of antibiotic exposures increased, the adjusted mean BMI-for-age *z* scores and likelihood of obesity increased significantly.

**Meaning:**

As repeated antibiotic exposure is a potentially modifiable risk factor for childhood obesity, interventions designed to reduce overprescribing of antibiotics in primary care could examine whether reducing overprescribing decreases the prevalence of childhood obesity.

## Teaser

This cohort study examines the association between repeated antibiotic exposure by age 48 months and body mass index at 54 months.

## Introduction

New Zealand has the third highest prevalence of obesity among Organisation for Economic Co-operation and Development countries.^1^ Pediatric obesity is associated with development of cardiovascular risk factors in later life, such as type 2 diabetes, hypertension, dyslipidemia, and metabolic syndrome.^2^ Antibiotic exposures in early life can change the bacterial composition of the intestine (microbiome), potentially increasing the risk of childhood obesity.^3^ Antibiotics affect weight by altering gut microbiota, which moderates the harvesting, storage, and expenditure of energy from dietary sources.^3^

Several child cohort studies have reported associations of antibiotic exposure with higher body mass index (BMI),^4,5^ overweight,^5,6,7^ or obesity.^8,9,10^ However, previous studies addressing this issue have limitations, including parent-reported antibiotic exposure,^5,6,10^ parent-reported weight and height,^11^ lack of height data,^12^ use of covariates measured at the same point as BMI,^7^ and restricting antibiotic exposure to children with specific diagnoses.^13,14^ Furthermore, previous studies were not designed to incorporate antibiotic exposure during pregnancy.

The overall aim of the present analysis was to examine the association between antibiotic exposure by age 48 months and body mass at age 54 months, using a cohort that has established linkage to antibiotic prescribing data and collected reliably measured anthropometric data. Specifically, this research evaluates whether the number, timing (age), and type of antibiotic exposures are associated with a higher body mass and an increased likelihood of overweight and obesity. This work incorporates antibiotic exposure during pregnancy.

## Methods

### Data Source and Sample

This analysis was undertaken during 2017 to 2018 within a prospective cohort study in New Zealand (the Growing Up in New Zealand study) in which 6853 children born during 2009 to 2010 were enrolled antenatally. Birth characteristics of the cohort aligned with the national birth cohort from 2007 to 2010. The Ministry of Health Northern Y Regional Ethics Committee approved the study, and enrolled parents gave written informed consent. The Data Access Committee that was established for this cohort study approved access to data sets used in this analysis. A detailed profile of the cohort is described elsewhere.^15^ Reporting of this analysis followed the Strengthening the Reporting of Observational Studies in Epidemiology (STROBE) reporting guideline.

Parents completed computer-assisted personal interviews antenatally and when their children were aged approximately 9, 24, and 54 months. Of the 6156 children who participated in the 54-month follow-up, 5734 of 6156 (93%) had both weight and height measured in duplicate by trained interviewers using standard protocols and identical sets of calibrated equipment. Where duplicate measurements of height differed by more than 1 cm or weight by more than 500 g, a third measurement was taken. The study obtained community pharmacy antibiotic dispensing data from the New Zealand Pharmaceutical Collection database for the 5666 children whose parents consented to external data linkage in addition to their mothers’ antibiotic dispensing data during pregnancy. Systemic use of antibacterial agents is via prescription only in New Zealand.

Antenatal, perinatal, and 24-month follow-up questionnaires were reviewed for availability of data to assess factors that could affect body mass. To exclude children with antenatal conditions affecting growth, the *International Statistical Classification of Diseases and Related Health Problems, Tenth Revision (ICD-10)* codes Q00 to Q99 were used to determine the presence of congenital anomalies.

The Figure shows the exclusion procedure used to select the study sample. This included 5406 singletons with prescription data and 54-month weight and height measurements and excluded 278 singletons who met 1 or more of the following criteria: gestational age less than 28 weeks or missing data, birth weight data missing, or congenital anomalies present. This resulted in an analytic sample of 5128 children.

**Figure.**
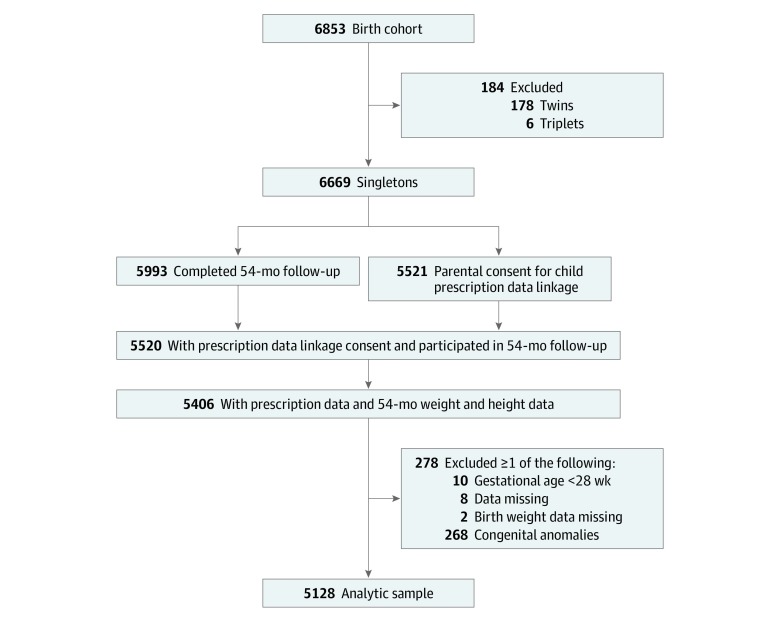
Selection of the Study Population From the Growing Up in New Zealand Cohort

### Measures of Body Mass

Two measures of body mass were derived using the child’s weight and height measurements, age when measured, and sex. We derived BMI-for-age *z* scores (*z*BMI) using World Health Organization child growth chart standards.^16^ Using the child’s BMI (calculated as weight in kilograms divided by height in meters squared), age, and sex, the second measure was derived using the International Obesity Task Force (IOTF) cutoff points for overweight and obesity defined to pass through adult BMI values of 25 and 30, respectively.^17^ The IOTF definition is less arbitrary and was developed to encourage direct comparisons of childhood obesity trends worldwide. The IOTF BMI variable categorizes children into 3 groups (normal or underweight, overweight, and obese).

### Measures of Antibiotic Exposure

Variables measuring children’s exposure to any antibiotics and to 4 types (penicillins, macrolides, cephalosporins, and co-trimoxazole) between birth and age 48 months were derived to quantify the following: any exposure (yes or no), number of dispensings, age at first exposure, and timing (age) of exposures. Maternal exposure to antibiotics during pregnancy was quantified as any exposure (yes or no) and number of dispensings between the estimated date of conception and child’s date of birth.

### Statistical Analysis

The unadjusted analysis of 5128 participants examined associations of measures of antibiotic exposure with continuous variables (*z*BMI and potential confounders) using generalized linear models and categorical variables (IOTF BMI and potential confounders) using χ^2^ tests. In addition, unadjusted associations between any exposure to antibiotics (yes or no) and potential confounders were examined. Continuous variables were summarized using their means and standard deviations, and categorical variables were summarized using counts and percentages. Owing to study ethical requirements, “<10” denotes categories with fewer than 10 participants.

The multivariable analysis sample comprised 4398 children with complete (nonmissing) data for factors significantly associated (*P* < .05) with body mass and excluded variables with missing data for at least 10% of the analytic sample (maternal relationship status, smoking in pregnancy, and self-reported prepregnancy BMI). Missing data for self-reported prepregnancy BMI were due to a higher proportion of missing values for self-reported height (9%) than weight (4%). Therefore, self-reported weight was added to the multivariable analysis and, because it had missing values for 3% of the multivariable sample (129 of 4398), the analysis commenced with multiple imputation (25 replicates) that included the following variables: log value of self-reported weight (assumed to be missing at random), any antibiotic exposure, and factors significantly associated with body mass in the unadjusted analysis.

Using the imputed data set, multivariable generalized linear models were used to assess adjusted associations between antibiotic exposure variables and *z*BMI (continuous outcome); results were summarized using pooled adjusted means and their standard errors. For the IOTF BMI variable (nominal outcome), multinomial logistic regression was used to determine whether antibiotic exposure was associated with increased likelihood of overweight or obesity with normal and underweight children as the comparison group; results were summarized using pooled adjusted odds ratios (aORs) and their 95% confidence intervals. Multivariable models adjusted for the child’s sex; birth weight; delivery mode; birth season; birth order; antireflux medication dispensed by age 48 months; sleep duration at age 24 months; time spent (last weekday) watching television, DVDs, or videos at age 24 months; exclusive breastfeeding duration; and dietary intake at age 24 months. The models also adjusted for maternal age, self-identified ethnicity, education, socioeconomic deprivation,^18^ self-reported prepregnancy weight (log scale), antibiotic exposure in pregnancy, and alcohol use in pregnancy. Statistical analyses were performed using SAS software version 9.4 (SAS Institute Inc). A 2-tailed *P* < .05 was considered statistically significant.

## Results

### Study Sample

The 5128 singletons (2622 [51%] male) had a mean (SD) birth weight of 3527 (542) g. Among mothers (mean [SD] age, 30.4 [5.8] years), 3012 of 5119 (59%) had less than a bachelor’s degree, 2098 of 5116 (41%) were of non-European ethnicities, and 1289 of 5125 (25%) lived in households in the highest quintile of area-level deprivation at enrollment.

### Selection of the Study Population

Of 6669 singletons, 5521 (83%) had maternal consent for child prescription data (Figure). Compared with singletons whose mothers provided this consent, singletons lacking consent had lower birth weight (mean [SD], 3477 [541] g vs 3520 [553] g) but did not differ with respect to gestational age, delivery mode, and sex (eTable 1 in the Supplement). In addition, uniparous mothers who did not provide consent for child prescription data linkage were younger (mean [SD], 28.4 [6.2] vs 30.4 [5.9] years) and disproportionally had less than a bachelor’s degree (838 [73%] vs 3258 [60%]), were of non-European ethnicities (780 [68%] vs 2259 [41%]), and lived in households in the highest quintile of area-level deprivation (435 [38%] vs 1400 [25%]).

Among singletons whose mothers provided consent for child prescription data linkage, 98% (5405 of 5520) had both weight and height measured at the 54-month follow-up (Figure). Singletons with weight and height data did not differ significantly from those without these data with respect to birth weight, gestational age, delivery mode, and maternal age, education, ethnicity, and socioeconomic deprivation (eTable 2 in the Supplement). However, singletons lacking weight and height data were disproportionally male (71 [62%] vs 2791 [52%]).

The 4398 children included in the multivariable analyses did not differ significantly from the 730 children excluded owing to incomplete data with respect to birth weight, gestational age, delivery mode, and sex (eTable 3 in the Supplement). However, the excluded group had mothers who, at childbirth, were younger (mean [SD] age, 29.0 [6.2] vs 30.6 [5.7] years) and disproportionally had less than a bachelor’s degree (504 [70%] vs 2508 [57%]), were of non-European ethnicities (443 [62%] vs 1655 [38%]), and lived in households in the highest quintile of area-level deprivation (287 [39%] vs 1002 [23%]).

### Factors Associated With Any Antibiotic Exposure by Age 48 Months

Of 5128 children in the analytic sample, 4886 (95%) were exposed to antibiotics by age 48 months. Compared with the unexposed, a larger proportion of children in the exposed group were male (2523 [52%] vs 99 [41%]), had been prescribed antireflux medications by age 48 months (771 [16%] vs 17 [7%]), had breastfed for less than 3 months (1747 [37%] vs 64 [27%]), and had consumed 3 or more servings per day of soft drinks, snacks, and fast food at age 24 months (1939 [41%] vs 73 [31%]) (eTable 4 and eTable 5 in the Supplement). The exposed group had mothers who at childbirth were younger (mean [SD] age, 30.3 [5.8] vs 31.7 [5.7] years) and disproportionally had less than a bachelor’s degree (2908 [60%] vs 104 [43%]), were of non-European ethnicity (2041 [42%] vs 57 [24%]), and lived in households in the highest quintile of area-level deprivation (1252 [26%] vs 37 [15%]).

### Unadjusted Associations of Antibiotic Exposure With Body Mass

As shown in Table 1, children exposed to antibiotics had a higher *z*BMI (mean [SD], 0.95 [1.20] vs 0.65 [1.02]), which also increased significantly with the number of antibiotic dispensings (dose-response association). Compared with the unexposed, mean (SD) *z*BMI was also higher among children whose first antibiotic course was dispensed in the first year of life (1.05 [1.25] vs 0.65 [1.02]) but not for those whose exposure commenced after age 12 months (0.78 [1.09] vs 0.65 [1.02]). These associations were significant in separate analyses for boys and girls (eTable 6 in the Supplement). Of 5128 children in the analytic sample, 437 (9%) had obesity at age 54 months. A larger proportion of children with obesity (428 [98%]) were exposed to antibiotics than normal-weight or underweight children (3448 [95%]) (Table 1). Significant trends were present for the increase in proportion of children with overweight (χ^2^ *P* < .001 for trend) or obesity (χ^2^ *P* < .001 for trend) as the number of dispensings increased and age of first exposure decreased.

**Table 1.** Unadjusted Associations of Any Antibiotic Exposure by Age 48 Months With Body Mass at Age 48 to 68 Months

Variable	WHO Standard			IOTF Guideline			
	Participants, No. (%)^a^	BMI *z* Score (n = 5128)		Participants, No. (%)^a^			*P* Value
		Mean (SD)	*P* Value	Underweight or Normal Weight (n = 3640)	Overweight (n = 1051)	Obese (n = 437)	
Unexposed by age ≤48 mo	242 (5)	0.65 (1.02)	[Reference]	192 (5)	41 (4)	<10 (2)	
Exposed by age ≤48 mo	4886 (95)	0.95 (1.20)	<.001	3448 (95)	1010 (96)	428 (98)	.004
No. of dispensings for any antibiotics by age ≤48 mo							
1-3	1137 (22)	0.73 (1.05)	.32	889 (24)	193 (18)	55 (13)	<.001
4-6	1152 (22)	0.89 (1.09)	.005	829 (23)	239 (23)	84 (19)	
7-9	904 (18)	0.98 (1.24)	<.001	620 (17)	202 (19)	82 (19)	
10-12	601 (12)	1.03 (1.20)	<.001	420 (12)	116 (11)	65 (15)	
13-15	412 (8)	1.08 (1.35)	<.001	267 (7)	99 (9)	46 (11)	
>15	680 (13)	1.26 (1.40)	<.001	423 (12)	161 (15)	96 (22)	
Age at first exposure, mo							
0-6	1235 (24)	1.15 (1.29)	<.001	805 (22)	292 (28)	138 (32)	<.001
>6 to 12	1926 (38)	0.98 (1.22)	<.001	1337 (37)	403 (38)	186 (43)	
>12 to 18	844 (16)	0.81 (1.14)	.07	627 (17)	160 (15)	57 (13)	
>18 to 24	391 (8)	0.83 (1.00)	.06	288 (8)	85 (8)	18 (4)	
>24 to 30	201 (4)	0.68 (0.95)	.79	161 (4)	30 (3)	10 (2)	
>30 to 36	132 (3)	0.76 (1.24)	.40	104 (3)	20 (2)	<10 (2)	
>36 to 42	90 (2)	0.66 (1.07)	.94	74 (2)	11 (1)	<10 (1)	
>42 to 48	67 (1)	0.73 (1.24)	.63	52 (1)	<10 (1)	<10 (1)	
Timing of exposure							
Unexposed at age ≤12 mo but exposed at age >12 mo	1725 (34)	0.78 (1.09)	.10	1306 (36)	315 (30)	104 (24)	<.001
Exposed at age ≤12 mo with or without subsequent exposure	3161 (62)	1.05 (1.25)	<.001	2142 (59)	695 (66)	324 (74)	

Abbreviations: BMI, body mass index (calculated as weight in kilograms divided by height in meters squared); IOTF, International Obesity Task Force; WHO, World Health Organization.

^a^ Percentages may not add up to 100% owing to rounding.

Children exposed to and with increasing dispensings for penicillins, macrolides, cephalosporins, or co-trimoxazole had higher mean *z*BMI compared with no type-specific exposure (eTable 7 and eTable 8 in the Supplement). Associations between age at first exposure and increased *z*BMI were present in children whose exposure to penicillin began at age 0 to 6 months and greater than 6 to 12 months. For macrolides, cephalosporins, and co-trimoxazole, children whose exposure began in the first year of life had a significantly higher *z*BMI, but this association was also apparent for exposures initiated up to age 36 months.

### Unadjusted Associations of Perinatal, Environmental, and Social Factors With Body Mass

Several factors were associated with both a higher mean *z*BMI and likelihood of overweight or obesity (Table 2 and Table 3). Child factors included sex; birth weight; delivery mode; birth season; birth order; antireflux medication dispensed by age 48 months; sleep duration at age 24 months; time spent (last weekday) watching television, DVDs, or videos at age 24 months; exclusive breastfeeding duration; and dietary intake at age 24 months. Maternal factors were age, relationship status, ethnicity, education, socioeconomic deprivation, self-reported prepregnancy weight, self-reported diabetes before or during pregnancy, and alcohol use and antibiotic exposure during pregnancy.

**Table 2.** Perinatal Factors Potentially Associated With Body Mass at Age 48 to 68 Months

Variable	WHO Standard, BMI *z* Score		IOTF Guidelines			
	Mean (SD)	*P* Value	Participants, No. (%)^a^			*P* Value
			Underweight or Normal Weight	Overweight	Obese	
Total sample (n = 5128)	5128		3640	1051	437	
Sex (n = 5128)						
Female	0.86 (1.17)	<.001	1720 (47)	553 (53)	233 (53)	.001
Male	1.02 (1.22)		1920 (53)	498 (47)	204 (47)	
Birth weight, g (n = 5128)						
<3000	0.59 (1.22)	<.001	565 (16)	100 (10)	43 (10)	<.001
3000 to <3500	0.82 (1.16)		1282 (35)	299 (28)	114 (26)	
≥3500	1.11 (1.19)		1793 (49)	652 (62)	280 (64)	
Mode of delivery (n = 5114)						
Spontaneous vaginal delivery	0.95 (1.20)	<.001	2446 (67)	723 (69)	299 (69)	.001
Cesarean (planned or unplanned)	0.99 (1.21)		798 (22)	253 (24)	105 (24)	
Other assisted (ventouse or forceps)	0.74 (1.12)		388 (11)	72 (7)	30 (7)	
Season of birth (n = 5128)						
Summer (December-February)	0.99 (1.13)	.001	1060 (29)	329 (31)	129 (30)	.09
Autumn (March-May)	1.03 (1.25)		574 (16)	186 (18)	73 (17)	
Winter (June-August)	0.84 (1.24)		911 (25)	221 (21)	93 (21)	
Spring (September-November)	0.92 (1.19)		1095 (30)	315 (30)	142 (32)	
Parity (n = 5124)						
First child	0.87 (1.17)	.001	1558 (43)	413 (39)	159 (36)	.01
Previous pregnancy	0.99 (1.22)		2080 (57)	636 (61)	278 (64)	
Antibiotic dispensings during pregnancy (n = 5018)						
None	0.85 (1.15)	<.001	2407 (68)	616 (60)	239 (56)	<.001
1	1.01 (1.24)		753 (21)	251 (25)	106 (25)	
2	1.20 (1.25)		269 (8)	101 (10)	54 (13)	
≥3	1.33 (1.39)		136 (4)	55 (5)	31 (7)	
Maternal smoking before or during pregnancy (n = 4636)						
Continued smoking during pregnancy	1.39 (1.25)	<.001	254 (8)	107 (11)	81 (20)	<.001
Stopped smoking during pregnancy	1.34 (1.33)		257 (8)	111 (12)	70 (18)	
Nonsmokers	0.84 (1.15)		2793 (84)	716 (77)	247 (62)	
Maternal drinking before or during pregnancy (n = 5114)						
Any drinking during pregnancy	0.98 (1.16)	.15	1058 (29)	296 (28)	130 (30)	.002
Stopped drinking during pregnancy	0.91 (1.12)		1626 (45)	478 (46)	158 (36)	
Nondrinkers	0.95 (1.35)		947 (26)	273 (26)	148 (34)	
Self-reported diabetes before or during pregnancy (n = 5120)						
No	0.93 (1.18)	<.001	3511 (97)	1004 (96)	400 (92)	<.001
Yes	1.23 (1.23)		126 (3)	42 (4)	37 (8)	
Self-reported prepregnancy weight (n = 4947)						
Tertile 1 (≤60 kg)	0.56 (1.02)	<.001	1406 (40)	214 (22)	59 (14)	<.001
Tertile 2 (61 to 73 kg)	0.85 (1.05)		1244 (35)	330 (33)	94 (23)	
Tertile 3 (≥74 kg)	1.38 (1.38)		893 (25)	451 (45)	256 (63)	
Maternal age, y (n = 5126)						
<25	1.21 (1.25)	<.001	548 (15)	230 (22)	115 (26)	<.001
25-29	0.93 (1.15)		849 (23)	268 (26)	100 (23)	
30-34	0.83 (1.17)		1258 (35)	308 (29)	117 (27)	
≥35	0.92 (1.20)		985 (27)	243 (23)	105 (24)	
Maternal relationship status (n = 4640)						
Has a partner	0.91 (1.18)	<.001	3180 (96)	889 (95)	352 (89)	<.001
No partner	1.40 (1.37)		130 (4)	46 (5)	43 (11)	
Maternal ethnicity (n = 5116)						
European/New Zealander	0.82 (1.05)	<.001	2298 (63)	551 (53)	169 (39)	<.001
Māori	1.32 (1.20)		399 (11)	188 (18)	93 (21)	
Pacific	1.75 (1.38)		282 (8)	220 (21)	137 (31)	
Asian, Middle Eastern, Latin American, African, or other	0.43 (1.17)		652 (18)	89 (8)	38 (9)	
Maternal education (n = 5119)						
Secondary school or NCEA 1-4 or less	1.13 (1.23)	<.001	924 (25)	360 (34)	175 (40)	<.001
Diploma, trade certificate, or NCEA 5-6	1.05 (1.30)		1053 (29)	332 (32)	168 (38)	
Bachelor’s degree	0.75 (1.09)		982 (27)	222 (21)	54 (12)	
Higher degree	0.69 (1.00)		675 (19)	134 (13)	40 (9)	
Socioeconomic deprivation, New Zealand Deprivation Index quintile (n = 5125)^b^						
1 (Least deprived)	0.73 (0.93)	<.001	700 (19)	155 (15)	34 (8)	<.001
2	0.81 (1.14)		749 (21)	182 (17)	58 (13)	
3	0.78 (1.09)		695 (19)	164 (16)	54 (12)	
4	0.94 (1.23)		743 (20)	200 (19)	102 (23)	
5 (Most deprived)	1.30 (1.35)		752 (21)	348 (33)	189 (43)	

Abbreviations: BMI, body mass index (calculated as weight in kilograms divided by height in meters squared); IOTF, International Obesity Task Force; NCEA, National Certificates of Educational Achievement; WHO, World Health Organization.

^a^ Percentages may not add up to 100% owing to rounding.

^b^ The 2006 New Zealand Deprivation Index describes the deprivation experienced by groups of people in small areas (approximately a block in size in a city) using census data on income, home ownership, social support, employment status, educational status, living space, communication (access to a phone), and transportation (access to a car).

**Table 3.** Environmental and Social Factors Potentially Associated With Body Mass at Age 48 to 68 Months

Variable	WHO Standard, BMI *z* Score		IOTF Guidelines			
	Mean (SD)	*P* Value	Participants, No. (%)^a^			*P* Value
			Underweight or Normal Weight	Overweight	Obese	
Total sample (n = 5128)	5128		3640	1051	437	
Antireflux medication dispensed by age ≤48 mo (n = 5128)						
No	0.96 (1.19)	.001	3034 (83)	920 (88)	386 (88)	<.001
Yes	0.81 (1.20)		606 (17)	131 (12)	51 (12)	
Lifestyle factors at 24 mo						
Sleep duration, day and night, h (n = 5001)						
<11	1.26 (1.39)	<.001	328 (9)	133 (13)	86 (20)	<.001
11-14	0.90 (1.16)		3030 (85)	842 (83)	316 (75)	
>14	0.83 (1.19)		201 (6)	44 (4)	21 (5)	
Watching television, DVDs, or videos, weekday, h (n = 4929)						
0	0.81 (1.12)	<.001	839 (24)	183 (18)	73 (18)	<.001
<1	0.81 (1.04)		829 (24)	217 (22)	56 (14)	
1 to <2	0.93 (1.20)		947 (27)	285 (28)	117 (28)	
2 to <3	1.07 (1.17)		512 (15)	174 (17)	83 (20)	
≥3	1.19 (1.43)		386 (11)	144 (14)	84 (20)	
Feeding and dietary patterns						
Exclusive breastfeeding, mo (n = 4960)						
None	1.33 (1.61)	<.001	91 (3)	35 (3)	21 (5)	<.001
<1	0.92 (1.17)		491 (14)	147 (15)	64 (15)	
1-2	1.08 (1.26)		628 (18)	227 (23)	107 (26)	
3-4	0.94 (1.19)		966 (27)	262 (26)	120 (29)	
≥5	0.82 (1.11)		1359 (38)	336 (33)	106 (25)	
Diet, servings/d at age 24 mo						
Fruit, including dried fruit (n = 4956)						
First tertile (≤3)	0.92 (1.19)	.03	1367 (39)	396 (39)	161 (39)	.79
Second tertile (>3 to 4)	0.88 (1.16)		1118 (32)	302 (30)	123 (30)	
Third tertile (>4)	0.99 (1.20)		1048 (30)	313 (31)	128 (31)	
Vegetables (n = 4958)						
First tertile (<3)	1.00 (1.23)	.003	1183 (33)	376 (37)	162 (39)	.01
Second tertile (3 to <4)	0.87 (1.16)		1592 (45)	398 (40)	168 (40)	
Third tertile (≥4)	0.95 (1.18)		759 (21)	233 (23)	87 (21)	
Milk, cheese, yogurt (n = 4956)						
First tertile (≤3)	0.94 (1.21)	.34	1192 (34)	364 (36)	144 (34)	.01
Second tertile (>3 to <5)	0.91 (1.13)		1306 (37)	369 (36)	126 (30)	
Third tertile (≥5)	0.97 (1.26)		1026 (29)	279 (28)	150 (36)	
Bread, rice, pasta, cereal (n = 4949)						
First tertile (≤5)	0.88 (1.15)	.001	1201 (34)	330 (33)	130 (31)	.24
Second tertile (>5 to <7)	0.92 (1.17)		1385 (39)	388 (38)	155 (37)	
Third tertile (≥7)	1.04 (1.27)		938 (27)	292 (29)	130 (31)	
Spreads (n = 4955)^b^						
First tertile (≤2)	0.92 (1.20)	.17	1741 (49)	512 (51)	198 (47)	.59
Second tertile (>2 to 3)	0.91 (1.16)		902 (26)	248 (25)	101 (24)	
Third tertile (>3)	0.99 (1.20)		885 (25)	250 (25)	118 (28)	
Meat, protein alternatives, eggs (n = 4963)						
First tertile (<3)	0.91 (1.21)	.04	1507 (43)	390 (38)	165 (39)	<.001
Second tertile (3)	0.91 (1.13)		915 (26)	269 (27)	84 (20)	
Third tertile (>3)	1.00 (1.22)		1107 (31)	356 (35)	170 (41)	
Soft drinks, snacks, fast food (n = 4960)						
First tertile (<3)	0.81 (1.09)	<.001	2222 (63)	545 (54)	181 (44)	<.001
Second tertile (3)	1.05 (1.30)		778 (22)	252 (25)	118 (29)	
Third tertile (>3)	1.18 (1.30)		542 (15)	210 (21)	112 (27)	

Abbreviations: BMI, body mass index (calculated as weight in kilograms divided by height in meters squared); IOTF, International Obesity Task Force; WHO, World Health Organization.

^a^ Percentages may not add up to 100% owing to rounding.

^b^ Category includes butter; margarine (canola-, sunflower-, olive oil–, and rice bran–oil based); butter-margarine blends; jam, marmalade, and honey; peanut butter and hazelnut spread; and Vegemite and Marmite.

### Association Between Repeated Antibiotic Exposure and Body Mass

In multivariable analyses, antibiotic exposure was associated with both measures of body mass (Table 4). Adjusted mean (SE) *z*BMI increased significantly with the number of antibiotic dispensings for 4 to 6, 7 to 9, and more than 9 dispensings (unexposed, 0.87 [0.09]; 1-3 exposures, 0.92 [0.06] [*P* = .57]; 4-6 exposures, 1.06 [0.06] [*P* = .02]; 7-9 exposures, 1.06 [0.06] [*P* = .02]; >9 exposures, 1.08 [0.05] [*P* = .01]). Compared with those who were unexposed, children with 4 or more antibiotic dispensings had a higher adjusted mean *z*BMI, and receiving more than 9 dispensings was associated with greater likelihood of obesity (aOR, 2.41; 95% CI, 1.07-5.41). In addition, the increase in *z*BMI associated with 2 or more antibiotic dispensings during pregnancy remained statistically significant compared with no exposure (*z*BMI adjusted mean [SE], 1.08 [0.07] vs 0.96 [0.05]; *P* = .02). Stratified by sex, girls (but not boys) with 9 or more dispensings had a significantly higher adjusted mean (SE) *z*BMI than those unexposed (1.00 [0.08] vs 0.74 [0.12]; *P* = .02), but there was no association with overweight or obesity (aOR, 1.65; 95% CI, 1.00-2.71; *P* = .05) (eTable 9 in the Supplement).

**Table 4.** Multivariable Models of Antibiotic Exposure by Age 48 Months With Body Mass at Age 48 to 68 Months

Measures of Antibiotic Exposure by Age ≤48 mo^a^	Participants, No.	WHO Standard, BMI *z* Score (n = 4398)		IOTF Guidelines (n = 4398)^b^			
				Overweight (n = 873)		Obese (n = 354)	
		Adjusted Mean (SE)^a^	*P* Value	Adjusted OR (95% CI)^a^	*P* Value	Adjusted OR (95% CI)^a^	*P* Value
**Any Antibiotics**							
Any antibiotics by age ≤48 mo, No. of dispensings							
None	204	0.87 (0.09)	[Reference]	1 [Reference]	[Reference]	1 [Reference]	[Reference]
1-3	1007	0.92 (0.06)	.57	0.95 (0.63-1.46)	.83	1.11 (0.48-2.57)	.81
4-6	997	1.06 (0.06)	.02	1.36 (0.90-2.07)	.15	1.85 (0.81-4.23)	.14
7-9	784	1.06 (0.06)	.02	1.38 (0.90-2.13)	.14	2.05 (0.90-4.70)	.09
>9	1406	1.08 (0.05)	.01	1.23 (0.81-1.86)	.34	2.41 (1.07-5.41)	.03
Timing of exposure to any antibiotics							
Unexposed by age ≤48 mo	204	0.89 (0.09)	[Reference]	1 [Reference]	[Reference]	1 [Reference]	[Reference]
Unexposed at ≤12 mo but exposed at >12 mo	1522	1.02 (0.06)	.10	1.18 (0.79-1.78)	.42	1.69 (0.75-3.82)	.20
Exposed at ≤12 mo with or without subsequent exposure	2672	1.06 (0.05)	.03	1.21 (0.81-1.81)	.36	1.90 (0.86-4.23)	.11
**Type of Antibiotics**							
Penicillins, No. of dispensings							
None by age ≤48 mo	286	0.96 (0.08)	[Reference]	1 [Reference]	[Reference]	1 [Reference]	[Reference]
1-3	1411	0.96 (0.06)	.96	0.95 (0.67-1.34)	.77	0.95 (0.51-1.78)	.87
4-6	1138	1.04 (0.06)	.22	1.10 (0.77-1.56)	.61	1.34 (0.71-2.50)	.37
>6	1563	1.09 (0.05)	.04	1.11 (0.79-1.58)	.55	1.61 (0.87-2.98)	.13
Macrolides, No. of dispensings							
None by age ≤48 mo	3072	1.02 (0.05)	[Reference]	1 [Reference]	[Reference]	1 [Reference]	[Reference]
1	732	1.10 (0.06)	.08	1.16 (0.94-1.43)	.17	1.24 (0.90-1.70)	.19
≥2	594	1.09 (0.06)	.14	1.09 (0.86-1.37)	.49	1.61 (1.18-2.21)	.003
Cephalosporins, No. of dispensings							
None by age ≤48 mo	2968	1.02 (0.05)	[Reference]	1 [Reference]	[Reference]	1 [Reference]	[Reference]
1	758	1.07 (0.06)	.29	1.19 (0.97-1.47)	.10	1.11 (0.81-1.51)	.52
≥2	672	1.08 (0.06)	.22	1.10 (0.88-1.38)	.38	1.24 (0.90-1.70)	.19
Co-trimoxazole, No. of dispensings							
None by age ≤48 mo	2793	1.00 (0.05)	[Reference]	1 [Reference]	[Reference]	1 [Reference]	[Reference]
1	794	1.09 (0.06)	.05	1.05 (0.85-1.30)	.64	1.60 (1.18-2.17)	.002
≥2	811	1.11 (0.06)	.01	1.19 (0.97-1.45)	.10	1.52 (1.13-2.04)	.006

Abbreviations: BMI, body mass index (calculated as weight in kilograms divided by height in meters squared); IOTF, International Obesity Task Force; OR, odds ratio; WHO, World Health Organization.

^a^ Separate multivariable models were used for each measure. Each model adjusted for child’s sex; birth weight; delivery mode; birth season; birth order; antireflux medication dispensed by age 48 months; sleep duration at age 24 months; time spent (last weekday) watching television, DVDs, or videos at age 24 months; duration of exclusive breastfeeding; and dietary intake at age 24 months. Each model also adjusted for maternal age, ethnicity, education, socioeconomic deprivation, self-reported prepregnancy weight (log scale), and alcohol use and antibiotic exposure during pregnancy.

^b^ Multinomial logistic regression models with normal-weight or underweight children (n = 3171) as the reference group.

### Association Between Timing (Age) of Antibiotic Exposure and Body Mass

The multivariable analyses showed that antibiotic exposure beginning in the first year of life was associated with higher *z*BMI compared with no exposure (mean [SE], 1.06 [0.05] vs 0.89 [0.09]; *P* = .03), whereas antibiotic exposure that commenced after the first year of life was not (1.02 [0.06] vs 0.89 [0.09]; *P* = .10) (Table 4). Timing of exposure was not associated with overweight or obesity in the multivariable analysis.

### Association Between Type-Specific Antibiotic Exposure and Body Mass

By antibiotic type, the multivariable analysis showed that receiving more than 6 penicillin dispensings was associated with higher *z*BMI (mean [SE], 1.09 [0.05] vs 0.96 [0.08]; *P* = .04), as was receiving 2 or more co-trimoxazole dispensings (1.11 [0.06] vs 1.00 [0.05]; *P* = .01) compared with no type-specific exposure (Table 4). In addition, receiving 2 or more dispensings of macrolides was associated with obesity (aOR, 1.61; 95% CI, 1.18-2.21), as was receiving 2 or more dispensings of co-trimoxazole (aOR, 1.52; 95% CI, 1.13-2.04). Neither was associated with overweight. Exposure to cephalosporins was not associated with body mass in the multivariable analysis.

## Discussion

In this prospective cohort study that examined the association between antibiotic exposure by age 48 months and body mass at age 54 months, repeated exposure to antibiotics was associated with higher mean *z*BMI and greater likelihood of obesity. Antibiotic exposure during pregnancy was also associated with an increased *z*BMI. In addition, children whose antibiotic exposure began in the first year of life had a higher *z*BMI than those who were unexposed, whereas children whose antibiotic exposure commenced after the first year of life did not differ significantly from those who were unexposed. This would be expected, as children whose exposure commences earlier in life tend to have more antibiotic dispensings over time than those whose initial exposure is delayed.

To our knowledge, this is the first study to incorporate antibiotic exposure during pregnancy in examining the association between antibiotic exposures in early childhood and body mass. Antibiotic exposure in pregnancy was associated with higher BMI in children whose mothers had 2 or more antibiotic prescriptions compared with no exposure (adjusted mean *z*BMI difference of 0.12), but there was no association with overweight or obesity (aOR, 1.65; 95% CI, 1.00-2.71; *P* = .05). Previous cohort studies^19,20^ examining prenatal antibiotic exposure and childhood obesity did not include the children’s own antibiotic exposure. Mor et al^20^ reported a higher adjusted prevalence ratio for overweight and obesity in Danish children aged 7 to 16 years whose mothers had 3 or more antibiotic prescriptions during pregnancy compared with the unexposed. In a US study^19^ of 436 mother-child dyads, children whose mothers self-reported antibiotic use in the second and third trimesters had higher *z*BMI and an increased risk of obesity at 7 years. Findings from the present study suggest that antibiotic exposure both before and after birth are associated with body mass in early childhood.

Consistent with the present study, findings from cohort studies^4,9^ show that children whose antibiotic prescriptions are initiated during the first 6 or 12 months of life have higher BMI on average. These findings are consistent with the notion that gut microbiota are susceptible to disruptions, particularly during infancy when the founding microbiota infants acquire at birth have not developed into a stable microbial community.^3^ Such disruptions can occur via infant exposure to antibiotics and possibly via maternal antibiotic exposure, as infants acquire part of their early life microbiota from their mother.^3^

Several cohort studies^4,7,8,9^ have reported findings comparable to the present study with regard to repeated antibiotic exposure and body mass. A retrospective cohort study in the United Kingdom^8^ found an increasing likelihood of obesity at age 4 years with 3 or more antibiotic exposures by age 2 years. Among children attending a primary care practice network in the United States, those with 4 or more antibiotic prescriptions at age 0 to 23 months had an increased risk of obesity at age 24 to 59 months.^9^ In a Canadian birth cohort,^7^ children with antibiotic exposure in the first year of life were 1.7 and 2.6 times more likely to be overweight at ages 9 and 12 years, respectively. A population-based study^4^ in Finland reported higher *z*BMI at age 24 months and older in children exposed to antibiotics when aged younger than 24 months and a linear trend in *z*BMI with increasing antibiotic prescriptions.

Other cohort studies have reported either no association between antibiotic exposure and body mass or an association of unlikely clinical significance. A cohort study in the United States^14^ that used health care data reported a modest association of antibiotic exposure at ages younger than 2 years with *z*BMI and overweight or obesity prevalence at age 5 years; the authors concluded that the clinical significance of a 0.02 to 0.07 increase in BMI z-score at age 5 years is likely negligible for individual patients. However, misclassification of antibiotic exposure is possible, as the study used electronic records (as opposed to prescription dispensing or claims) only within, but not outside, participating health care networks. Misclassification of exposures and/or outcomes also potentially explains null findings in some cohort studies. For example, a study in the Netherlands^11^ found no association of any antibiotic exposure or type and timing of exposure with *z*BMI in children, but it assessed height and weight via parental report. A study in the United States^12^ reported no association between antibiotic exposure in the first 6 months of life and rate of weight change at ages 2 to 5 years, but the study lacked height measurements, used clinical measures of weight collected at 30 primary care practices, and did not measure antibiotics prescribed outside of the primary care network. Another US study^13^ concluded that there was no association between antibiotic exposure in infancy and BMI up to age 18 years even though it was restricted to those with clinical diagnoses of infections in infancy. Excluding children with antibiotic exposure because they lacked an infection diagnosis (instead of assessing exposure irrespective of the reason for it) contributes to exposure misclassification.

Some cohort studies^4,9,21,22^ have reported findings comparable to the present study with regard to exposure to specific antibiotic types and BMI. Differences by antibiotic type may be due to their different modes of action on bacteria. For instance, penicillins and cephalosporins target the bacterial cell wall by interrupting cell wall synthesis during cell division, macrolides target the ribosome by interfering with protein synthesis, and sulphonamides act by inhibiting folic acid synthesis.^21^ Bailey et al^9^ found a greater risk for obesity at age 24 to 59 months in children who at ages 0 to 23 months had 4 or more treatment episodes with broad-spectrum antibiotics (defined as systemic antibiotics excluding penicillin and amoxicillin), but not for narrow-spectrum antibiotics (penicillin and amoxicillin). Saari et al^4^ reported that boys (but not girls) with 2 or more macrolide prescriptions by age 24 months were more likely to be overweight at age 24 months or older and have higher *z*BMI on average. Comparably, Korpela et al^22^ found an association between exposure to macrolide-type antibiotics during the previous 2 years and BMI in children aged 2 to 7 years. Furthermore, intestinal microbiota in the macrolide-exposed children showed changes previously associated with increased BMI, adiposity, obesity, or metabolic diseases in children or adults.^22^

### Strengths and Limitations

This study has several strengths. It used dispensed prescription data to determine children’s antibiotic exposure at the same age to allow an equal opportunity for exposure, as well as maternal exposure during pregnancy; however, the national pharmaceutical database does not include medications prescribed in hospitals. In addition, this study examined the number, timing (age), and type (penicillins, macrolides, cephalosporins, co-trimoxazole) of antibiotic exposure. The study outcome measures were based on reliably measured BMI data in a large and ethnically diverse sample and the analyses accounted for many factors associated with body mass.

This study also has some limitations. First, mothers who did not consent to prescription data linkage and whose children had incomplete data in the multivariable analysis were disproportionally of non-European ethnicities and lived in more deprived areas. As these groups have higher obesity prevalence rates in New Zealand, this limitation may have underestimated the magnitude of the association between antibiotic exposure and body mass. Second, maternal household income was unreported by 24% of enrolled mothers (1613 of 6822); hence, it was not used in the analysis. Third, maternal BMI and gestational weight gain data could not be obtained via maternal health records. Fourth, prepregnancy weight and height were collected via self-report. A study on the validity of self-reported height, weight, and BMI by the US Centers for Disease Control and Prevention^23^ showed a reporting bias in these data whereby women tended to underreport their weight and overreport their height, resulting in an underestimation of BMI; underreporting of weight increased as BMI and age increased.

## Conclusions

This study found that repeated exposure to antibiotics by age 4 years was associated with significantly higher BMI and greater likelihood of obesity at age 4.5 years. In addition, repeated antibiotic exposure in pregnancy was associated with higher BMI in young children. Timing of antibiotic exposure was also associated with BMI; compared with those who were unexposed, children whose exposure began during the first year of life had higher mean *z*BMI , whereas those whose exposure began after the first year of life did not. The study findings suggest that repeated antibiotic exposure may be a potentially modifiable risk factor for childhood obesity. Future research could examine whether interventions that seek to reduce unnecessary prescribing of antibiotics to children in primary care (for example, clinical rules that identify children at low risk of needing antibiotics^24^) can also reduce the prevalence of childhood obesity.

## Notes

Supplement. **eTable 1.** Characteristics of Singleton Children (n=6669) by Whether Maternal Consent to Child Prescription Data Linkage Was Granted **eTable 2.** Characteristics of Singletons Who Participated in the 54-Month Follow-up and Had Prescription Data Linkage Consent (n=5520) by Whether Weight and Height Were Measured at Age 54 Months **eTable 3.** Characteristics of Singleton Children in the Analytic Sample (n=5128) by Whether They Were Excluded From Multivariable Analyses Due to Missing Data **eTable 4.** Perinatal Factors Potentially Associated With Antibiotic Exposure by Age ≤48 Months **eTable 5.** Environmental and Social Factors Potentially Associated With Antibiotic Exposure by Age ≤48 Months **eTable 6.** Sex-Stratified Unadjusted Associations of Any Antibiotic Exposure by Age ≤48 Months With Body Mass at Age 48-68 Months **eTable 7.** Unadjusted Associations of Exposure to Penicillins by Age ≤48 Months With Body Mass at Age 48-68 Months (n=5128) **eTable 8. **Unadjusted Associations of Exposure to Macrolides, Cephalosporins and Co-trimoxazole by Age ≤48 Months With Body Mass at Age 48-68 Months **eTable 9.** Sex-Stratified Multivariable Analysis of Antibiotic Exposure by Age 48 Months With Body Mass at Age 48-68 Months
