# Supplementary material for: Association of Repeated Antibiotic Exposure Up to Age 4 Years With Body Mass at Age 4.5 Years
Source: JAMA Netw Open. 2020 Jan 22;3(1):e1917577. doi: 10.1001/jamanetworkopen.2019.17577 (PMC6991235; doi:10.1001/jamanetworkopen.2019.17577)
Supplement: Supplement. — eTable 1. Characteristics of Singleton Children (n=6669) by Whether Maternal Consent to Child Prescription Data Linkage Was Granted eTable 2. Characteristics of Singletons Who Participated in the 54-Month Follow-up and Had Prescription Data Linkage Consent (n=5520) by Whether Weight and Height Were Measured at Age 54 Months eTable 3. Characteristics of Singleton Children in the Analytic Sample (n=5128) by Whether They Were Excluded From Multivariable Analyses Due to Missing Data eTable 4. Perinatal Factors Potentially Associated With Antibiotic Exposure by Age ≤48 Months eTable 5. Environmental and Social Factors Potentially Associated With Antibiotic Exposure by Age ≤48 Months eTable 6. Sex-Stratified Unadjusted Associations of Any Antibiotic Exposure by Age ≤48 Months With Body Mass at Age 48-68 Months eTable 7. Unadjusted Associations of Exposure to Penicillins by Age ≤48 Months With Body Mass at Age 48-68 Months (n=5128) eTable 8. Unadjusted Associations of Exposure to Macrolides, Cephalosporins and Co-trimoxazole by Age ≤48 Months With Body Mass at Age 48-68 Months eTable 9. Sex-Stratified Multivariable Analysis of Antibiotic Exposure by Age 48 Months With Body Mass at Age 48-68 Months [file jamanetwopen-3-e1917577-s001.pdf]

Supplementary Online Content

Chelimo C, Camargo CA Jr, Morton SMB, Grant CC. Association of repeated antibiotic exposure up to age 4 years with body mass at age 4.5 years. *JAMA Netw Open*. 2020;3(1):e1917577. doi:10.1001/jamanetworkopen.2019.17577

eTable 1. Characteristics of Singleton Children (n=6669) by Whether Maternal Consent to Child Prescription Data Linkage Was Granted
eTable 2. Characteristics of Singletons Who Participated in the 54-Month Follow-up and Had Prescription Data Linkage Consent (n=5520) by Whether Weight and Height Were Measured at Age 54 Months
eTable 3. Characteristics of Singleton Children in the Analytic Sample (n=5128) by Whether They Were Excluded From Multivariable Analyses Due to Missing Data
eTable 4. Perinatal Factors Potentially Associated With Antibiotic Exposure by Age ≤48 Months
eTable 5. Environmental and Social Factors Potentially Associated With Antibiotic Exposure by Age ≤48 Months
eTable 6. Sex-Stratified Unadjusted Associations of Any Antibiotic Exposure by Age ≤48 Months With Body Mass at Age 48-68 Months
eTable 7. Unadjusted Associations of Exposure to Penicillins by Age ≤48 Months With Body Mass at Age 48-68 Months (n=5128)
eTable 8. Unadjusted Associations of Exposure to Macrolides, Cephalosporins and Co-trimoxazole by Age ≤48 Months With Body Mass at Age 48-68 Months
eTable 9. Sex-Stratified Multivariable Analysis of Antibiotic Exposure by Age 48 Months With Body Mass at Age 48-68 Months

This supplementary material has been provided by the authors to give readers additional information about their work.

© 2020 Chelimo C et al. *JAMA Network Open.*

eTable 1. Characteristics of singleton children (n=6669) by whether maternal consent to child prescription data linkage was granted

	Maternal consent to prescription data linkage		
**Variable**	**No**	**Yes**	
	**(n=1148)**	**(n=5521)**	
	**n (%)[a]**	**n (%)[a]**	**p-value**
**Child factors**			
Birthweight (grams) (n=6659) [mean (SD)]	3477 (541)	3520 (553)	0.02
Gestational age (WHO criteria) (n=6652)			
Extremely preterm (<28 weeks)	<10 (0)	<10 (0)	0.34
Very preterm (28 - <32 weeks)	<10 (0)	28 (1)	
Moderate to late preterm (32 - <37 weeks)	44 (4)	245 (4)	
Term (37+ weeks)	1092 (96)	5235 (95)	
Mode of delivery (n=6599)			
Spontaneous vaginal delivery	763 (69)	3678 (67)	0.21
Caesarean (planned/unplanned)	245 (22)	1287 (23)	
Other assisted (ventouse/forceps)	92 (8)	534 (10)	
Sex (n=6669)			
Male	590 (51)	2863 (52)	0.78
Female	558 (49)	2658 (48)	
**Maternal factors**			
Maternal age (years) (n=6665) [mean (SD)]	28.4 (6.2)	30.4 (5.9)	<0.001
Maternal age (years) (n=6665)			
<25	329 (29)	966 (18)	<0.001
25-29	312 (27)	1318 (24)	
30-34	282 (25)	1794 (33)	
≥35	224 (20)	1440 (26)	
Maternal ethnicity (n=6646)			
European / New Zealander	364 (32)	3243 (59)	<0.001
Māori	200 (17)	728 (13)	
Pacific	282 (25)	692 (13)	
Asian	240 (21)	742 (13)	
MELAA / Other	58 (5)	97 (2)	
Maternal education (n=6646)			
Sec school/NCEA 1-4 or less	479 (42)	1579 (29)	<0.001
Diploma/Trade cert/NCEA 5-6	359 (31)	1679 (31)	
Bachelor's degree	171 (15)	1333 (24)	
Higher degree	132 (12)	914 (17)	
Social deprivation (NZDep quintiles) (n=6663)			
1 (least deprived)	129 (11)	949 (17)	<0.001
2	139 (12)	1074 (19)	
3	172 (15)	970 (18)	
4	271 (24)	1124 (20)	
5 (most deprived)	435 (38)	1400 (25)	

MELAA=Middle Eastern / Latin American / African, NCEA=National Certificates of Educational Achievement, NZDep=New Zealand Deprivation Index, SD=standard deviation

[a] Percentages may not add up to 100% due to rounding

© 2020 Chelimo C et al. *JAMA Network Open.*

**eTable 2. Characteristics of singletons who participated in the 54-month follow-up and had prescription data linkage consent (n=5520) by whether weight and height were measured at age 54 months**			
	**Child's weight and height measured at the 54 month follow-up**		
**Variable**	**No**	**Yes**	
	**(n=115)**	**(n=5405)**	
	**n (%)[a]**	**n (%)[a]**	**p-value**
**Child factors**			
Birthweight (grams) (n=5518) [mean (SD)]	3453 (699)	3521 (550)	0.19
Gestational age (WHO criteria) (n=5512)			
Preterm (<37 weeks)	10 (9)	268 (5)	0.07
Term (37+ weeks)	105 (91)	5129 (95)	
Mode of delivery (n=5498)			
Spontaneous vaginal delivery	69 (60)	3608 (67)	0.28
Caesarean (planned/unplanned)	32 (28)	1255 (23)	
Other assisted (ventouse/forceps)	14 (12)	250 (10)	
Sex (n=5520)			
Male	71 (62)	2791 (52)	0.03
Female	44 (38)	2614 (48)	
**Maternal factors**			
Maternal age (years) (n=5517) [mean (SD)]	30.5 (5.4)	30.4 (5.9)	0.86
Maternal age (years) (n=5517)			
<25	16 (14)	950 (18)	0.37
25-29	34 (30)	1284 (24)	
30-34	33 (29)	1761 (33)	
≥35	32 (28)	1407 (26)	
Maternal ethnicity (n=5501)			
European / New Zealander	64 (56)	3179 (59)	0.78
Māori	17 (15)	711 (13)	
Pacific	14 (12)	677 (13)	
Asian	19 (17)	723 (13)	
MELAA / Other	<10 (1)	96 (2)	
Maternal education (n=5504)			
Sec school/NCEA 1-4 or less	31 (27)	1548 (29)	0.29
Diploma/Trade cert/NCEA 5-6	43 (37)	1635 (30)	
Bachelor's degree	21 (18)	1312 (24)	
Higher degree	20 (17)	894 (17)	
Social deprivation (NZDep quintiles) (n=5516)			
1 (least deprived)	14 (12)	935 (17)	0.13
2	27 (23)	1047 (19)	
3	15 (13)	955 (18)	
4	21 (18)	1103 (20)	
5 (most deprived)	38 (33)	1367 (25)	
MELAA=Middle Eastern / Latin American / African, NCEA=National Certificates of Educational Achievement, NZDep=New Zealand Deprivation Index, SD=standard deviation			
[a] Percentages may not add up to 100% due to rounding			

© 2020 Chelimo C et al. *JAMA Network Open.*

eTable 3. Characteristics of singleton children in the analytic sample (n=5128) by whether they were excluded from multivariable analyses due to missing data

	Included in the multivariable analysis		
**Variable**	**No**	**Yes**	
	**(n=730)**	**(n=4398)**	
	**n (%)[a]**	**n (%)[a]**	**p-value**
**Child factors**			
Birthweight (grams) (n=5128) [mean (SD)]	3494 (548)	3533 (541)	0.08
Gestational age (WHO criteria) (n=5128)			
Preterm (<37 weeks)	36 (5)	210 (5)	0.85
Term (37+ weeks)	694 (95)	4188 (95)	
Mode of delivery (n=5114)			
Spontaneous vaginal delivery	505 (71)	2963 (67)	0.23
Caesarean (planned/unplanned)	146 (20)	1010 (23)	
Other assisted (ventouse/forceps)	65 (9)	425 (10)	
Sex (n=5128)			
Male	368 (50)	2254 (51)	0.67
Female	362 (50)	2144 (49)	
**Maternal factors**			
Maternal age (years) (n=5126) [mean (SD)]	29.0 (6.2)	30.6 (5.7)	<0.001
Maternal age (years) (n=5126)			
<25	188 (26)	705 (16)	<0.001
25-29	178 (24)	1039 (24)	
30-34	218 (30)	1465 (33)	
≥35	144 (20)	1189 (27)	
Maternal ethnicity (n=5116)			
European / New Zealander	275 (38)	2743 (62)	<0.001
Māori	130 (18)	550 (13)	
Pacific	167 (23)	472 (11)	
Asian	134 (19)	555 (13)	
MELAA / Other	12 (2)	78 (2)	
Maternal education (n=5119)			
Sec school/NCEA 1-4 or less	276 (38)	1183 (27)	<0.001
Diploma/Trade cert/NCEA 5-6	228 (32)	1325 (30)	
Bachelor's degree	140 (19)	1118 (25)	
Higher degree	77 (11)	772 (18)	
Social deprivation (NZDep quintiles) (n=5125)			
1 (least deprived)	85 (12)	804 (18)	<0.001
2	102 (14)	887 (20)	
3	97 (13)	816 (19)	
4	156 (21)	889 (20)	
5 (most deprived)	287 (39)	1002 (23)	

MELAA=Middle Eastern / Latin American / African, NCEA=National Certificates of Educational Achievement, NZDep=New Zealand Deprivation Index, SD=standard deviation

[a] Percentages may not add up to 100% due to rounding

© 2020 Chelimo C et al. *JAMA Network Open.*

eTable 4. Perinatal factors potentially associated with antibiotic exposure by age ≤48 months			
	Antibiotic exposure by age ≤48 months		
Variable	No	Yes	
	n (%)[a]	n (%)[a]	p-value
Total sample (n=5128)	242 (5)	4886 (95)	
Sex (n=5128)			
Female	143 (59)	2363 (48)	0.001
Male	99 (41)	2523 (52)	
Birthweight (grams) (n=5128) [mean (SD)]	3518 (457)	3528 (546)	0.78
Birthweight (grams) (n=5128)			
<3000	28 (12)	680 (14)	0.03
3000 to <3500	99 (41)	1596 (33)	
≥3500	115 (47)	2610 (53)	
Mode of delivery (n=5114)			
Spontaneous vaginal delivery	168 (69)	3300 (68)	0.18
Caesarean (planned/unplanned)	45 (19)	1111 (23)	
Other assisted (ventouse/forceps)	29 (12)	461 (9)	
Season of birth (n=5128)			
Summer (Dec–Feb)	71 (29)	1447 (30)	0.97
Autumn (Mar–May)	37 (15)	796 (16)	
Winter (Jun–Aug)	58 (24)	1167 (24)	
Spring (Sep–Nov)	76 (31)	1476 (30)	
Parity (n=5124)			
First child	112 (46)	2018 (41)	0.13
Previous pregnancy	130 (54)	2864 (59)	
Maternal smoking pre/during pregnancy (n=4636)			
Continue smoking in pregnancy	<10 (4)	434 (10)	0.001
Stopped smoking in pregnancy	13 (6)	425 (10)	
Non-smokers	201 (90)	3555 (80)	
Maternal drinking pre/during pregnancy (n=5114)			
Any drinking in pregnancy	72 (30)	1412 (29)	0.63
Stopped drinking in pregnancy	111 (46)	2151 (44)	
Non-drinkers	58 (24)	1310 (27)	
Maternal diabetes pre/during pregnancy (n=5120)			
No	238 (98)	4677 (96)	0.06
Yes	<10 (2)	201 (4)	
Self-reported pre-pregnancy weight (n=4947)			
1st Tertile (≤60 kilograms)	98 (42)	1581 (34)	0.02
2nd Tertile (61 to 73 kilograms)	79 (33)	1589 (34)	
3rd Tertile (≥74 kilograms)	59 (25)	1541 (33)	
Maternal age (years) (n=5126) [mean (SD)]	31.7 (5.7)	30.3 (5.8)	<0.001
Maternal age (years) (n=5126)			
<25	29 (12)	864 (18)	0.004
25-29	55 (23)	1162 (24)	
30-34	73 (30)	1610 (33)	
≥35	85 (35)	1248 (25)	
Maternal relationship status (n=4640)			
Has a partner	211 (95)	4210 (95)	0.87
No partner	11 (5)	208 (5)	
Maternal ethnicity (n=5116)			
European / New Zealander	184 (76)	2834 (58)	<0.001

© 2020 Chelimo C et al. *JAMA Network Open.*

Māori	16 (7)	664 (14)	
Pacific	13 (5)	626 (13)	
Asian / MELAA / Other	28 (12)	751 (15)	
Maternal education (n=5119)			
Sec school/NCEA 1-4 or less	44 (18)	1415 (29)	<0.001
Diploma/Trade cert/NCEA 5-6	60 (25)	1493 (31)	
Bachelor's degree	83 (34)	1175 (24)	
Higher degree	55 (23)	794 (16)	
Social deprivation (NZDep quintiles) (n=5125)			
1 (least deprived)	52 (21)	837 (17)	<0.001
2	58 (24)	931 (19)	
3	56 (23)	857 (17)	
4	39 (16)	1006 (21)	
5 (most deprived)	37 (15)	1252 (26)	

BMI=body mass index, MELAA=Middle Eastern / Latin American / African, NCEA=National Certificates of Educational Achievement, NZDep=New Zealand Deprivation Index, SD=standard deviation

[a] Percentages may not add up to 100% due to rounding

© 2020 Chelimo C et al. *JAMA Network Open.*

eTable 5. Environmental and social factors potentially associated with antibiotic exposure by age ≤48 months			
	Antibiotic exposure by age ≤48 months		
Variable	No	Yes	
	n (%)[a]	n (%)[a]	p-value
Total sample (n=5128)	242 (5)	4886 (95)	
Medication dispensed by age ≤48 months			
Antireflux medication (n=5128)			
No	225 (93)	4115 (84)	<0.001
Yes	17 (7)	771 (16)	
Lifestyle factors (at 24 months)			
Sleep duration (day and night) (n=5001)			
<11 hours	21 (9)	526 (11)	0.46
11-14 hours	203 (85)	3985 (84)	
>14 hours	15 (6)	251 (5)	
Watching TV, DVD, videos (weekday) (n=4929)			
0 hours	55 (23)	1040 (22)	0.12
<1 hour	66 (28)	1036 (22)	
1 to <2 hours	65 (27)	1284 (27)	
2 to <3 hours	29 (12)	740 (16)	
3+ hours	22 (9)	592 (13)	
Feeding and dietary patterns			
Exclusive breastfeeding (n=4960)			
Never breastfed	<10 (3)	140 (3)	0.02
<1 month	24 (10)	678 (14)	
1-2 months	33 (14)	929 (20)	
3-4 months	64 (27)	1284 (27)	
5+ months	106 (45)	1695 (36)	
Diet - servings per day (at 24 months)			
Fruit (includes dried fruit) (n=4956)			
1st Tertile (≤3)	81 (34)	1843 (39)	0.27
2nd Tertile (>3 to 4)	77 (32)	1466 (31)	
3rd Tertile (>4)	80 (34)	1409 (30)	
Vegetables (n=4958)			
1st Tertile (<3)	76 (32)	1645 (35)	0.43
2nd Tertile (3 to <4)	101 (43)	2057 (43)	
3rd Tertile (≥4)	59 (25)	1020 (22)	
Milk, cheese, yoghurt (n=4956)			
1st Tertile (≤3)	89 (37)	1611 (34)	0.44
2nd Tertile (>3 to <5)	87 (37)	1714 (36)	
3rd Tertile (≥5)	62 (26)	1393 (30)	
Bread, rice, pasta, cereal (n=4949)			
1st Tertile (≤5)	87 (36)	1574 (33)	0.46
2nd Tertile (>5 to <7)	94 (39)	1834 (39)	
3rd Tertile (≥7)	58 (24)	1302 (28)	
Spreads (n=4955)			
1st Tertile (≤2)	125 (52)	2326 (49)	0.68
2nd Tertile (>2 to 3)	59 (25)	1192 (25)	
3rd Tertile (>3)	56 (23)	1197 (25)	
Meat, protein alternatives, eggs (n=4963)			
1st Tertile (<3)	86 (36)	1976 (42)	0.20
2nd Tertile (3)	66 (28)	1202 (25)	

© 2020 Chelimo C et al. *JAMA Network Open.*

3rd Tertile (>3)	87 (36)	1546 (33)	
Soft drinks, snacks, fast food (n=4960)			
1st Tertile (<3)	164 (69)	2784 (59)	0.01
2nd Tertile (3)	42 (18)	1106 (23)	
3rd Tertile (>3)	31 (13)	833 (18)	
BMI=body mass index, SD=standard deviation			
[a] Percentages may not add up to 100% due to rounding			

© 2020 Chelimo C et al. *JAMA Network Open.*

eTable 6. Sex-stratified unadjusted associations of any antibiotic exposure by age ≤48 months with body mass at age 48-68 months

	Boys (n=2622)				Girls (n=2506)		
**Variable**		**BMI z-score**				**BMI z-score**	
	**n (%)[a]**	**Mean (SD)**	**p-value**		**n (%)[a]**	**Mean (SD)**	**p-value**
Unexposed by age ≤48 months	99 (4)	0.74 (1.11)	Ref		143 (6)	0.59 (0.95)	Ref
Exposed by age ≤48 months	2523 (96)	1.03 (1.22)	0.02		2363 (94)	0.87 (1.18)	0.004
Dispensings for any antibiotics by age ≤48 months							
1-3	548 (21)	0.82 (1.11)	0.54		589 (24)	0.65 (0.97)	0.55
4-6	559 (21)	0.98 (1.12)	0.07		593 (24)	0.80 (1.06)	0.05
7-9	471 (18)	1.03 (1.22)	0.03		433 (17)	0.92 (1.25)	0.003
10-12	326 (12)	1.00 (1.13)	0.07		275 (11)	1.08 (1.28)	<0.001
13-15	249 (10)	1.14 (1.42)	0.005		163 (7)	0.99 (1.24)	0.002
>15	370 (14)	1.37 (1.37)	<0.001		310 (12)	1.13 (1.42)	<0.001
Age at first exposure							
0-6 months	685 (26)	1.20 (1.26)	<0.001		550 (22)	1.08 (1.31)	<0.001
>6-12 months	1017 (39)	1.04 (1.22)	0.02		909 (36)	0.92 (1.21)	0.002
>12-18 months	415 (16)	0.93 (1.19)	0.17		429 (17)	0.69 (1.06)	0.35
>18-24 months	183 (7)	0.83 (0.93)	0.56		208 (8)	0.83 (1.06)	0.06
>24-30 months	91 (3)	0.69 (0.99)	0.74		110 (4)	0.68 (0.92)	0.54
>30-36 months	58 (2)	0.94 (1.54)	0.33		74 (3)	0.62 (0.91)	0.85
>36-42 months	47 (2)	0.75 (1.02)	0.97		43 (2)	0.56 (1.11)	0.91
>42-48 months	27 (1)	1.04 (1.54)	0.26		40 (2)	0.52 (0.95)	0.75
Timing of exposure (age in months)							
Unexposed at ≤12 months, but exposed at >12 months	821 (31)	0.87 (1.15)	0.31		904 (36)	0.70 (1.03)	0.27
Exposed at ≤12 months +/- subsequently	1702 (65)	1.11 (1.24)	0.004		1459 (58)	0.98 (1.25)	<0.001

BMI=body mass index, SD=standard deviation

[a] Percentages may not add up to 100% due to rounding

© 2020 Chelimo C et al. *JAMA Network Open.*

**eTable 7. Unadjusted associations of exposure to penicillins by age ≤48 months with body mass at age 48-68 months (n=5128)**			
**Variable**		**BMI z-score**	
	**n (%)[a]**	**Mean (SD)**	**p-value**
Unexposed by age ≤48 months	336 (7)	0.73 (1.03)	Ref
Exposed by age ≤48 months	4792 (93)	0.96 (1.21)	0.001
Dispensings by age ≤48 months			
1-3	1592 (31)	0.77 (1.09)	0.54
4- 6	1326 (26)	0.93 (1.16)	0.005
7-9	829 (16)	1.07 (1.31)	<0.001
10-12	489 (10)	1.12 (1.24)	<0.001
>12	556 (11)	1.25 (1.32)	<0.001
Age at first exposure			
0-6 months	1077 (21)	1.17 (1.29)	<0.001
>6-12 months	1857 (36)	0.98 (1.21)	<0.001
>12-18 months	860 (17)	0.85 (1.17)	0.10
>18-24 months	431 (8)	0.85 (1.07)	0.14
>24-30 months	231 (5)	0.74 (0.92)	0.91
>30-36 months	153 (3)	0.62 (1.09)	0.37
>36-42 months	102 (2)	0.71 (1.34)	0.94
>42-48 months	81 (2)	0.70 (1.23)	0.84

BMI=body mass index, SD=standard deviation

[a] Percentages may not add up to 100% due to rounding

© 2020 Chelimo C et al. *JAMA Network Open.*

eTable 8. Unadjusted associations of exposure to macrolides, cephalosporins and co-trimoxazole by age ≤48 months with body mass at age 48-68 months												
	**Macrolides**				**Cephalosporins**				**Co-trimoxazole**			
	**(n=5128)**				**(n=5128)**				**(n=5128)**			
**Variable**		**BMI z-score**				**BMI z-score**				**BMI z-score**		
	**n (%)[a]**	**Mean (SD)**	**p-value**		**n (%)[a]**	**Mean (SD)**	**p-value**		**n (%)[a]**	**Mean (SD)**	**p-value**	
Unexposed by age ≤48 months	3568 (70)	0.89 (1.18)	Ref		3453 (67)	0.90 (1.16)	Ref		3240 (63)	0.87 (1.12)	Ref	
Exposed by age ≤48 months	1560 (30)	1.06 (1.22)	<0.001		1675 (33)	1.03 (1.26)	<0.001		1888 (37)	1.07 (1.31)	<0.001	
Dispensings by age ≤48 months												
1	851 (17)	1.05 (1.17)	<0.001		886 (17)	1.02 (1.19)	0.007		936 (18)	1.02 (1.30)	<0.001	
≥2	709 (14)	1.08 (1.28)	<0.001		789 (15)	1.03 (1.33)	0.004		952 (19)	1.11 (1.32)	<0.001	
Age at first exposure												
0-6 months	125 (2)	1.02 (1.38)	0.24		119 (2)	1.17 (1.31)	0.02		98 (2)	1.24 (1.48)	0.002	
>6-12 months	244 (5)	1.10 (1.22)	0.007		381 (7)	1.08 (1.21)	0.004		436 (9)	1.15 (1.30)	<0.001	
>12-18 months	256 (5)	1.03 (1.23)	0.07		296 (6)	1.04 (1.38)	0.05		433 (8)	1.02 (1.35)	0.02	
>18-24 months	245 (5)	1.19 (1.35)	<0.001		234 (5)	0.84 (1.13)	0.47		278 (5)	1.06 (1.13)	0.008	
>24-30 months	223 (4)	1.11 (1.12)	0.008		166 (3)	1.12 (1.48)	0.02		191 (4)	1.00 (1.26)	0.14	
>30-36 months	206 (4)	1.13 (1.21)	0.005		179 (3)	0.93 (1.05)	0.75		179 (3)	1.14 (1.39)	0.003	
>36-42 months	135 (3)	0.88 (1.01)	0.94		169 (3)	1.05 (1.30)	0.11		162 (3)	1.02 (1.34)	0.11	
>42-48 months	126 (2)	0.88 (1.09)	0.91		131 (3)	1.02 (1.18)	0.26		111 (2)	0.87 (1.37)	0.96	
BMI=body mass index, SD=standard deviation												
[a] Percentages may not add up to 100% due to rounding												

© 2020 Chelimo C et al. *JAMA Network Open.*

eTable 9. Sex-Stratified Multivariable Analysis of Antibiotic Exposure by Age 48 Months With Body Mass at Age 48-68 Months

		WHO Standard (n=2144)			IOTF Guidelines (n=2144)[a]	
**Girls**		**BMI z-score**			**Overweight or obese (n=650)**	
		**Adjusted[b] mean**	**p-value**		**Adjusted[b] OR**	**p-value**
	**n**	**(SE)**			**(95% CI)**	
Any antibiotic dispensings by age ≤48 months						
None (unexposed)	127	0.74 (0.12)	Ref		1.00	Ref
1-3	526	0.81 (0.09)	0.51		1.04 (0.63-1.72)	0.89
4-6	507	0.91 (0.09)	0.11		1.49 (0.90-2.47)	0.12
7-9	373	0.96 (0.09)	0.05		1.68 (1.00-2.81)	0.05
>9	611	1.00 (0.08)	0.02		1.65 (1.00-2.71)	0.05
		**WHO Standard (n=2254)**			**IOTF Guidelines (n=2254)[a]**	
**Boys**		**BMI z-score**			**Overweight or obese (n=577)**	
		**Adjusted[b] mean**	**p-value**		**Adjusted[b] OR**	**p-value**
	**n**	**(SE)**			**(95% CI)**	
Any antibiotic dispensings by age ≤48 months						
None (unexposed)	77	0.99 (0.14)	Ref		1.00	Ref
1-3	481	1.02 (0.08)	0.83		0.85 (0.44-1.64)	0.63
4-6	490	1.19 (0.08)	0.14		1.35 (0.71-2.59)	0.36
7-9	411	1.18 (0.08)	0.20		1.29 (0.67-2.50)	0.44
>9	795	1.16 (0.07)	0.20		1.22 (0.64-2.32)	0.54

BMI=body mass index, IOTF=International Obesity Task Force, NZDep=New Zealand Deprivation Index, OR=odds ratio, SE=standard error, WHO=World Health Organization

[a] Multinomial logistic regression models with normal weight or underweight children as the reference group (n=1494 for girls; n=1677 for boys)

[b] Each model adjusted for child's sex, birthweight, mode of delivery, season of birth, birth order, anti-reflux medication dispensed by age ≤48 months, sleep duration at age 24 months, time spent (last weekday) watching TV/DVD/video at age 24 months, duration of exclusive breastfeeding, and dietary intake at age 24 months. Each model also adjusted for maternal age, ethnicity, education, socioeconomic deprivation, self-reported pre-pregnancy weight (log scale), antibiotic exposure in pregnancy, and alcohol use in pregnancy.

© 2020 Chelimo C et al. *JAMA Network Open.*
